# Telerehabilitation: A Solution for Patients After Hip Fracture?

**DOI:** 10.37825/2239-9747.1048

**Published:** 2024-03-21

**Authors:** Alessia Bramanti, Rossella Ciurleo, Carmine Vecchione, Andrea Turolla, Noemi Piramide, Michele Ciccarelli, Erica Piramide, Marina Garofano, Michele Senatore, Mariaconsiglia Calabrese

**Affiliations:** a Department of Medicine, Surgery and Dentistry “Medical School of Salerno”, University of Salerno, Italy; b IRCCS Centro Neurolesi Bonino Pulejo of Messina, Italy; c Department of Biomedical and Neuromotor Sciences, University of Bologna, Bologna, Italy; d IRCCS Azienda Ospedaliero-Universitaria di Bologna, Bologna, Italy; e Canopo Centro Studi, Salerno, Italy; f Department of Medicine, Surgery and Dentistry, University of Cagliari, Italy; g “Ordine dei Fisioterapisti” of Salerno, Italy; h Rehabilitation Department, A.O.U. “San Giovanni di Dio e Ruggi d’Aragona”, 84125, Salerno, Italy

**Keywords:** Hip fracture, Physiotherapy, Telerehabilitation, Remote rehabilitation

## Abstract

- Hip fracture is the most common cause of hospitalization in frail geriatric subjects due to osteoporosis and recurrent falls. The clinical practice guidelines for rehabilitation after surgery in patients with hip fractures recommend to start treatment early. However, the outbreak of SARS-CoV-2 pandemic between December 2019 and January 2020 forced to lockdown. Thus, telerehabilitation seemed the best solution to remote assistance.

In this scenario, the aim of our study is to assess the effects of telerehabilitation and to clarify and rearrange the knowledge about its usability and feasibility in patients after hip fracture in emergency conditions, such as the pandemic of SARS-CoV-2.

Three databases were systematically searched from caption to December 2023, considering only articles published in peer-reviewed journals, with the use of three macro-areas: ‘telerehabilitation’, ‘remote rehabilitation’ and ‘hip fracture’. In the present review, 26 articles were considered eligible and 10 were included.

Heterogeneous results were found due to the different characteristics of the patients recruited in the studies, designs and type of the studies, and reporting/conducting of the research. Also, the typologies of telerehabilitation provided were various.

In conclusion, this review demonstrated that telerehabilitation is safe, effective and well tolerated from patients and seems to be not inferior to the conventional physiotherapy. It also plays a positive role in psychological rehabilitation, in the prevention of complications and in the maintenance of achieved goals. However, further studies are needed to guide the clinical practice in providing the better posology and typology of telerehabilitation.

## 1. Introduction

The population worldwide is progressively growing older and there is the need to manage age-related diseases [1,2]. Hip fracture is the most common cause of hospitalization in frail geriatric subjects due to osteoporosis and recurrent falls [3]. Patients after hip fracture experience a reduction of functional independence, physical mobility, balance, walking ability and social participation [4,5]. Thus, the clinical practice guidelines for post-operative rehabilitation in patients with hip fractures recommend to start rehabilitation early, one week after the surgery if possible, with the aim to reestablish the previous fitness level [4]. Balance training and progressive resistance exercises are strongly recommended. Weight-bearing exercises, exercises aimed at restoring functional independence and early gait were also suggested [4].

However, the outbreak of SARS-CoV-2 pandemic between December 2019 and January 2020 forced worldwide population to lockdown, severe restriction and social distancing to better control the spread of the virus [6,7]. This emergency period has strongly stressed the international health services by limiting rehabilitation care-paths [6,7]. Physiotherapists and physicians worldwide tried to manage not only the consequences of COVID-19, but also out-patients needing rehabilitation programs. Indeed, the World Physiotherapy and, particularly in our territories, the Italian Association of Physiotherapy warmly advised a long-distance support for the patients.

In this scenario, telerehabilitation seemed the best solution for remote assistance, as a rehabilitation tool providing long-distance support to patients at home [6].

Telerehabilitation was already used in the past years as a supportive or substitutive instrument for usual care in patients with musculoskeletal and neurological conditions, with positive results [8]. Lately, several studies have demonstrated the effectiveness of home-based telerehabilitation programs, specific for neurological issues, even by synchronous combination of telemedicine with virtual reality [6,9,10].

Indeed, telerehabilitation has the advantage of reaching the patients at home, eventually in rural situation, leading to reduction of costs, times, physical barriers and caregivers’ burden [6,9].

However, the information on telerehabilitation in patients after hip fractures are heterogeneous. The aim of this review is to assess the effects of telerehabilitation and to clarify and rearrange the knowledge about its usability and feasibility in patients after hip fracture in emergency conditions, such as the pandemic of SARS-CoV-2.

## 2. Methodology

### 2.1. Inclusion and exclusion criteria

In this review, we included articles written in English language and with available full-text, and studies targeting only humans and focused on telerehabilitation of patients after hip fracture. Studies including animal models or written in other languages were excluded.

### 2.2. Search strategy

PubMed database, Physiotherapy Evidence Database (PEDro) and Scopus were systematically searched from caption till December 2023, taking into account only articles published in peer-reviewed journals, with the following search strategy: Virtual rehabilitation OR Telerehabilitation OR telerehabilitation OR Telemedicine OR remote rehabilitation OR Telerehab AND hip fracture for PubMed and Scopus; for PEDro database we used the simple search with the following strings: virtual rehabilitation AND hip fracture; telerehabilitation AND hip fracture; telerehabilitation AND hip fracture; telemedicine AND hip fracture; remote rehabilitation AND hip fracture; telerehab AND hip fracture.

**Fig. 1:**
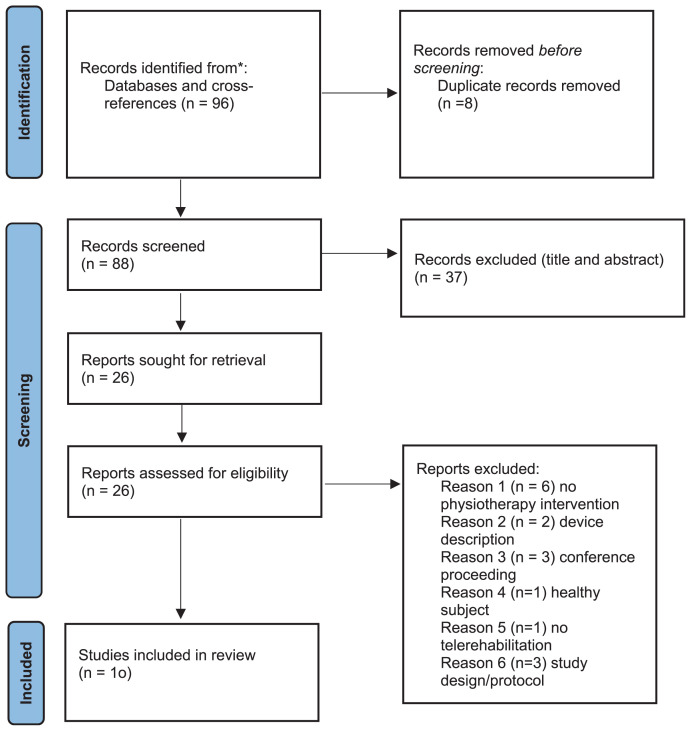
PRISMA flowchart showing the number of records identified, the included and excluded studies, and the reasons for exclusions.

No restrictions on publication date were placed and 96 articles were identified from the databases searching. Eight Duplicate records were removed with EndNote. All articles were screened for title/ abstract and full-text by two independent reviewers, separately selected and discussed for conflict about doubtful cases on the inclusion/ exclusion criteria. After the screening phase, 10 articles were considered eligible for fulfilling the study aim and were included and discussed in the present review (Fig. 1). All the studies were checked for the PEDro score **(**Table 1**)** to evaluate the research quality, the methodological quality and evidence level of the included studies [11]. A study scored as 6 or more is considered having good methodological quality (6–8: good; 9–10: excellent) and scored as 5 or less is considered being of acceptable or poor quality (4–5: acceptable; <4: poor) [12].

## 3. Results

In the present review, 10 articles were considered eligible and were included. All articles are summarized in Table 2. Heterogeneous results were found due to the different characteristics of the patients recruited in the studies (eg., age, gender, occupation, life habits etc.), designs and type of studies, and reporting/conducting of the research.

The typologies of telerehabilitation provided were various: videos, videos combined with written/real-time instructions, real-time videoconference, wearable sensors and apps.

Many studies considered only the telerehabilitation approach (experimental group) [13–16], while others compared telerehabilitation with conventional physiotherapy, or telephone follow-up as control groups [17–19]. Moreover, in many cases only telerehabilitation was proposed to patients [13–16], while in others a combination of telerehabilitation and face-to-face physical therapy was compared with conventional physiotherapy [20,21]. There was no consensus in the rehabilitation posology (both for telematic and conventional modalities), that ranged from 30 days to 12 weeks of training period, or from daily training to 5 times of rehabilitation sessions per week as session frequency, or from 30 to 90 min as session duration. Wu et al., finally, did not report any information regarding the exercise posology [18].

### 3.1. Telerehabilitation

As mentioned above, many authors proposed only telerehabilitation to patients as treatment modality [13–16]. One of these used interactive systems [14], two videos and instructions through an app and one a real-time videoconference system [15,16]. Bedra and colleagues [14] used the Home Automated Telemanagement (HAT) system, a home server able to follow patients in their exercise program and to send information on performance to the physiotherapist’s server. Findings from this studies were positive. Their approaches resulted feasible, safe and effective for use in a home-based rehabilitation setting. Adherence of the patients was high and significant improvements in the functional outcomes were reported [14]. On the other hand, Jensen et al. proposed the “My Hip Fracture Journey” App containing pictographs, videos, illustrated exercises, and written information [15]. The ability to perform self-care and autonomy in the activity of daily living were promoted by this app. Yuan–Yuan Zhang et al. provided wearable devices, including infrared thermometers, blood pressure and heart rate meters, available to both patients and their family members, for regular detection of vital signs at home, to be transmitted at physician desktop by Internet of Things (IoT) technologies [17].

Finally, Jorgensen and his group used a real-time videoconference telerehabilitation program immediately after discharge from hospital after hipfractur [13]. In their study, they included 9 patients, but 7 withdrew due to fatigue and fragile situation after hospitalization, as well as due to skills needed for managing the telerehabilitation program, thus results were not reliable.

The posology of all the studies were different **(see**Table 2**)**.

### 3.2. Telerehabilitation combined with conventional physiotherapy

Only two studies considered the combination of telerehabilitation with face-to-face physical therapy [20,21]. Kalron and colleagues [20] presented a video platform software program with lower limb exercises for the experimental group and an exercise booklet for the control group. All the patients joined also conventional physiotherapy. The program included 6 weeks of training, 3 times per week, 40–50 min each session. Clinical improvements were reported in both groups, but they were greater in the telerehabilitation group, particularly in the mobility tasks.

Li and colleagues [21] proposed to the patients the Caspar Health e-system program which included videos, pictures and written/verbal instructions for exercises execution. Patients in the control group received the same program of exercises at home, but via paper-and-pencil instructions. The frequency and duration of patient’s home program was not reported. All the patients attended also 90 min of conventional occupational therapy training, twice a week, for three weeks, in a day hospital setting. Authors found improvements in fall efficacy and physical performance in the experimental group and a better muscle strength in the control group.

### 3.3. Telerehabilitation versus conventional physiotherapy

Three studies compared telerehabilitation with conventional rehabilitation [17,18,22].

The research group of Ortiz-Pina published a study protocol [23] and a subsequent study [22] on the @ctivehip protocol training consisting of on-demand videos and written instructions for activities and exercises for 12 weeks, 5 times per week per 50–60 min each. The experimental protocol was compared with conventional home face-to-face therapy. Authors found higher functional independence and physical performance in the experimental group. Mora-Traverso et al. [19] also used the activehip protocol in comparison with a control group undergoing in-person home rehabilitation. The @ctivehip telerehabilitation program seems to be a promising treatment to improve the quality of life, psychological factors and promotes recovery of previous fitness level. Two other studies showed that a telerehabilitation program was more effective than the traditional one, because it allowed a better continuity of treatment and early prevention of possible complications [17,18].

## 4. Discussion

This review was aimed to explore the current literature on telerehabilitation in patients after hip fracture and its feasibility in emergency conditions, such as the pandemic of SARS-CoV-2.

The knowledge in the field of telerehabilitation is still uncertain but current findings are in favor of its safety and good acceptance from patients, with not inferior effects than conventional physiotherapy.

Internet-based rehabilitation management system provides a new approach for implementing rehabilitation at home to elderly patients after hip fracture. Telerehabilitation allows patients to receive continuous treatment, making it universally available, easier for patients access, thus mastering the key points of rehabilitation. In addition, the Internet-based rehabilitation management system can be applied remotely to the participant’s home.

## 5. Conclusion

However, we can conclude that Telerehabilitation is a valuable tool in addition to conventional therapy and offers a safe and effective alternative to traditional in-person care, especially during emergency periods, as seen with COVID-19. Telerehabilitation seems to promote the physical rehabilitation of patients, but it also plays a positive role in psychological rehabilitation, prevention of complications and the maintenance of achieved goals.

## Figures and Tables

**Table 1:** summary of PEDro scores for included studies

	1	2	3	4	5	6	7	8	9	10	11	TOTAL SCORE	QUALITY
Bedra et al. (2015)	Y	N	N	Y	N	N	N	Y	Y	Y	Y	6	Good
Jensen et al. (2019)	Y	N	N	Y	N	N	N	N	Y	N	N	3	Poor
Jørgensen et al. (2021)	Y	N	N	Y	N	N	N	N	N	N	N	2	Poor
Kalron et al. (2018)	Y	Y	Y	N	Y	N	N	Y	Y	Y	Y	8	Good
Li et al. (2022)	Y	Y	Y	Y	N	N	Y	Y	Y	Y	Y	9	Excellent
Mora-Traverso et al. (2022)	Y	N	N	Y	N	Y	Y	Y	Y	Y	Y	8	Good
Ortiz-Piña et al. (2021)	Y	N	N	Y	N	Y	Y	Y	Y	Y	Y	8	Good
Prieto-Moreno et al. (2022)	Y	N	N	Y	N	N	N	Y	N	Y	Y	5	Acceptable
Wu et al. (2023)	Y	N	N	Y	N	N	Y	Y	Y	Y	Y	7	Good
Zhang et al. (2022)	Y	Y	N	Y	N	N	Y	Y	Y	Y	Y	8	Good

1: inclusion criteria and source, 2: random allocation, 3: concealed allocation, 4: similarity at baseline, 5: subject blinding, 6: therapist blinding, 7: assessor blinding, 8: completeness of follow up, 9: intention-to-treat analysis, 10: between-group statistical comparisons, 11: point measures and variability.

**Table 2:** Eligible articles included in the present review

References	Study type	Experimental group	Control group	No training group	Outcomes	Results
Bedra et al.	Quasi-experimental study	(N = 10) Exercises using a Home Automated Telemanagement (HAT) system 1 session per day 30 days of training	NA	NA	Functional independence General health Emotional status of the patients Functional impairment of the lower extremities Physical performance Patient satisfaction Cognitive status of the patient Confidence of the patient to exercises Quality of life Acceptance/feasibility of the telerehabilitation system	Statistically significant improvements were found in: Exercise self-efficacy, Mobility, Quality of life, Patient satisfaction on telerehabilitation after the training period. Telerehabilitation is feasible, safe and effective in older adults post hip fracture.
Jensen et al.	Qualitative study	(N = 15) Exercises using an app (videos + instructions) 3–4 weeks of training The details about the number and the duration of the sessions are not known	NA	NA	Functional independence Physical performance Mobility The issue of getting old Usability of the tablet and app Emotional status of the patients	Patients showed improvements in functional independence and satisfaction on telerehabilitation.
Jørgensen et al.	Feasibility study	(N = 2) Exercises using an a real-time videoconference system 3 times per week 45 min each session 4 weeks of training	NA	NA	Physical performance Mobility Fitness level Cognitive status of the patient	The sample size is too small to have noteworthy results.
Kalron et al.	Feasibility pilot study	(N = 15) Exercises using a video platform (videos + instructions) + face-to-face physical therapy sessions 2 times per week 3 times per week 40–50 min each session 6 weeks of training	(N = 17) Exercises with booklet instructions + face-to-face physical therapy sessions 2 times per week 3 times per week 40–50 min each session 6 weeks of training	NA	Physical performance Mobility Balance	Patients in the experimental group improved in all outcome measures relative to patients in the control group. Telerehabilitation combined with face-to-face physical therapy training is more effective than face-to-face physical therapy alone with booklet advices.
Li et al.	Randomized control trial	(N = 16) Exercises with videos, pictures and written and verbal instructions by app (+face-to-face occupational therapy at day hospital) 3 weeks of training	(N = 15) Exercises with paper-and-pencil instructions (+face-to-face occupational therapy at day hospital) 3 weeks of training	NA	Pain perception, Quadriceps strength Balance Walking speed Physical performance Fear of falling	No significant differences between the two groups were found: fall efficacy and physical performance better in the experimental group muscle strength better in the control group
Mora-Traverso et al.	non-randomized clinical trial	(N = 35) Home-based multidisciplinary telerehabilitation intervention of 12 weeks Five 50-to-60-minute online-based sessions per week (2 of occupational therapy and 3 of physical exercise) Content delivered through the @ctivehip online platform	(N = 36) Usual care and rehabilitation Between 5 and 15 sessions of home based in-person rehabilitation Recommendations and physical exercises to do at home	NA	Quality of life Psychological factors Fitness level	The @ctivehip telerehabilitation program seems to be a promising treatment to improve: The quality of life Psychological factors recover previous fitness level
Ortiz-Piña et al.	Non-randomized clinical trial	(N = 28) Exercises using an online platform (videos + instructions) 5 times per week 50–60 min each session 12 weeks of training	(N = 34) 5–15 post-discharge multidisciplinary rehabilitation sessions at home	NA	Functional independence Physical performance Balance Mobility	Telerehabilitation program of 12 weeks had better results in functional independence and physical performance relative to traditional home-based rehabilitation.
Prieto-Moreno et al.	feasibility international and multicentre study	(N = 36 Spain N = 33 Belgium) Health educational section composed of five modules for older adults and family caregivers, and Two additional specific modules for family caregivers Rehabilitation section: two sessions of physical exercise and one session of occupational therapy per week for 12 weeks. Through pre-recorded videos with a voice-over to describe each exercise.	NA	(N = 36 Spain N = 33 Belgium) training in the use of the ActiveHip + mHealth system 1 or 2 days before hospital discharge	Adoption, Usage, Satisfaction with the app Perceived quality of the app FIM SPPB NRS	The ActiveHip + mHealth system obtained satisfactory feasibility results in both countries. The intervention had positive effects on functional status, pain and physical fitness
Wu et al.	Quasi-experimental study	(N = 43) Patients established contact with the hospital through the hip fracture post-operative management system. They use a rehab box including a microcomputer and Bluetooth connect ab peripherals	(N = 42) Text version of the post-operative rehabilitation program. Patients are followed up weekly by telephone and their questions answered.	NA	The physical function of patients was assessed using HHS and FIM. Psychological recovery was assessed using SAS	HHS and FIM score were significantly higher in the telerehabilitation group than in the telephone group. The SAS score was significantly lower in the telerehabilitation group than in the telephone group. Complication rate was significantly lower in the telerehabilitation group.
Yuan–Yuan Zhang et al.	randomized controlled trial	(N = 29) Exercises using an application installed on a smartphone. The system automatically send rehabilitation videos and health information based on the patient’s condition and the number of days after surgery. Measurement of the patient’s vital signs at home, through wearable devices	(N = 29) the patients received telephone follow-ups at 2 weeks, 1 month, 2 months, and 3 months after discharge from the hospital Verbal inquiry and assessment to understand patients rehabilitation process, compliance, pain, ability to take care of themselves in daily life, Treatment and control of underlying diseases, and psychological and mental status	NA	HHS FIM TUG SPPB ADL	This Internet-based rehabilitation management system can improve: Functional recovery of the hip joint Enhance the ability to perform ADL Is simple to operate and easy to use.

**Abbreviations:** Number (N), Activity of Daily Living (ADL), Functional Independence Measure (FIM), Short Physical Performance Battery (SPPB), Harris Hip Score (HHS), Timed Up and Go (TUG), Numeric Rating Scale for Pain (NRS), Self-Rating Anxiety Scale (SAS).
